# Human Thromboxane A_2_ Receptor Genetic Variants: *In Silico*, *In Vitro* and “In Platelet” Analysis

**DOI:** 10.1371/journal.pone.0067314

**Published:** 2013-06-28

**Authors:** Scott Gleim, Jeremiah Stitham, Wai Ho Tang, Hong Li, Karen Douville, Prashen Chelikani, Jeffrey J.Rade, Kathleen A. Martin, John Hwa

**Affiliations:** 1 Internal Medicine, Cardiovascular Medicine, Yale University School of Medicine, New Haven Connecticut, United States of America; 2 Department of Pharmacology and Toxicology, Dartmouth Medical School, Hanover New Hampshire, United States of America; 3 Department of Oral Biology, University of Manitoba Faculty of Dentistry, Winnipeg, Manitoba, Canada; 4 Internal Medicine-Section of Cardiology, UMass School of Medicine and Medical Center, Worcester, Massachusetts, United States of America; University of North Dakota, United States of America

## Abstract

Thromboxane and its receptor have emerged as key players in modulating vascular thrombotic events. Thus, a dysfunctional hTP genetic variant may protect against (hypoactivity) or promote (hyperactivity) vascular events, based upon its activity on platelets. After extensive *in silico* analysis, six hTP-α variants were selected (C^68^S, V^80^E, E^94^V, A^160^T, V^176^E, and V^217^I) for detailed biochemical studies based on structural proximity to key regions involved in receptor function and *in silico* predictions. Variant biochemical profiles ranged from severe instability (C^68^S) to normal (V^217^I), with most variants demonstrating functional alteration in binding, expression or activation (V^80^E, E^94^V, A^160^T, and V^176^E). In the absence of patient platelet samples, we developed and validated a novel megakaryocyte based system to evaluate human platelet function in the presence of detected dysfunctional genetic variants. Interestingly, variant V80E exhibited reduced platelet activation whereas A160T demonstrated platelet hyperactivity. This report provides the most comprehensive *in silico*, *in vitro* and *“in platelet”* evaluation of hTP variants to date and highlightscurrent inherent problems in evaluating genetic variants, with possible solutions. The study additionally provides clinical relevance to characterized dysfunctional hTP variants.

## Introduction

Cardiovascular disease and events affect nearly 80 million Americans, costing over $280 billion annually [1]. Landmark papers [2], [3] have demonstrated that COX-derived prostanoids (prostacyclin and thromboxane) significantly influence cardiovascular disease, with thromboxane promoting atherothrombotic events. Diabetes mellitus [4] ^,^ [5], smoking [6], [7], [8], and obesity [9], [10] (key cardiovascular risk factors) are associated with increased thromboxane levels. Low dose aspirin, a cyclooxygenase inhibitor (COX-1>> COX-2), reduces platelet thromboxane production leading to protective effects in women (against strokes) and men (myocardial events) [11]. Moreover, selective COX-2 inhibitors suppress PGI_2_ production, abolishing its anti-atherothrombotic effect by allowing unrestricted thromboxane activity [2], [3]. These clinical findings have strong support from genetic deletion of the receptors for prostacyclin (IP) and thromboxane (TP) in mice [2], [3], [12]. Excess TxA_2_ signaling promote atherothrombosis.In contrast, deficient TxA2 signaling suppress atherothrombosis.

The human thromboxane A_2_ receptor (hTP) is a typical Class A rhodopsin-like G-protein coupled receptor. Despite being the first human eicosanoid receptor cloned [13], differential effects of hTP isoforms cloned from placenta (hTP alpha) and endothelium (hTP beta) [14] and their clinical importance highlight the need to better understand receptor structure and function [15] ^,^ [16]. Mutational analysis of this receptor has identified a number of residues influencing binding [17], effector coupling [18], stability [19], and ligand recognition [20]. Thromboxane receptor variants are implicated in asthma susceptibility and severity [21], [22], [23], atopic dermatitis [24], and an autosomal dominant bleeding disorder [25].

We (and others) have identified a growing number of naturally occurring variants of the hTP receptor. Using predictive commonly used *in silico* algorithms in conjunction with molecular modeling we selected six variants to biochemically study. We also developed a functional assay to assess for platelet function conferred by the mutation. We report that two variants V80E and A160T have opposing roles on platelet activity, inhibiting and promoting platelet activation, respectively. In addition to the presentation and validation of a novel platelet based assay for the study of genetic variants, we highlight the poor predictive nature of some of the current, commonly used predictive algorithms for genetic variant and possible solutions.

## Materials and Methods

### Prediction of Variant Influence

Six polymorphism effect prediction algorithms were assessed to provide a view of expected outcomes based on differing methodology. Each algorithm utilizes protein sequence homology to some degree, with some incorporating physicochemical properties of side-chains (ProPhylER and Mutation Taster) or including structural annotations (SNPs3D and SNAP). The SIFT (Sorting Intolerant From Tolerant) algorithm (http://blocks.fhcrc.org/sift/SIFT.html) [26] infers functional importance from sequence homology. Based on a PSI-BLAST search alignment, SIFT returns a scaled probability matrix for the likelihood of a protein to tolerate each of the twenty amino acids at each position in the protein. Output values for each amino acid change tolerance from SIFT ranges from 0 (damaging) to 1 (neutral). SNAP (screening for non-acceptable polymorphisms) (http://www.rostlab.org/services/SNAP) [27] incorporates SIFT predictions along with PSI-BLAST alignment conservation, while incorporating additional sequence information including solvent accessibility and secondary structure (PROF), flexibility (PROFbval), and annotations from PMD (Protein Mutant Database) and. As with SIFT, SNAP reports a tolerance range from 0 (damaging) to 1 (neutral). PolyPhen (PPH) (http://genetics.bwh.harvard.edu/pph/) [28] has been superseded by PolyPhen-2 (PPH2) (http://genetics.bwh.harvard.edu/pph2/). PolyPhen-2 provides two results based on HumVar (13,032 human disease causing mutations from UniProt and 8,946 human nonsynonymous single-nucleotide polymorphisms (nsSNPs) and HumDiv [29]. These prediction algorithms return a scaled probability from 0 (neutral) to >1 (damaging). The module of the SNPs3D resource (http://www.SNPs3D.org) [30] which focuses on predicting SNP influence on protein function, separately evaluates protein structural stability analysis and sequence conservation using a support vector machine approach trained on monogenic disease. ProPhylER (http://www.prophyler.org) [31] combines the physicochemical properties of amino acid side chains and the observed evolutionary variation of those properties to infer deleterious substitutions. The Grantham score [32] represents the original amino acid substitution scoring method, presenting a scale increasing from zero to indicate the degree of side-chain physicochemical difference between the original and mutant amino acid. Largely superseded by newer methodology, this value is still occasionally referenced, as shown by the most recently developed resource in this list, Mutation Taster. Mutation Taster (http://www.mutationtaster.org) [33] uses a naïve Bayes classifier to predict disease potential of an alteration. In addition, this method incorporates a prediction of potential changes in splice acceptor/donor probabilities (NNSplice).

### Homology Model Construction

The human thromboxane A_2_ receptor (hTP) sequence was aligned with solved crystal structures, bovine rhodopsin (OPSD, UniProt P02699) and the human beta2-adrenergic receptor (ADRB2, UniProt P0755) in ClustalW. Both the PAM250 and BLOSUM algorithms indicated hTP to align more closely with OPSD (similarity score 30.16) than with ADRB2 (33.48). Each bundle of seven transmembrane α-helices was therefore based on a 2.8Å crystallographic bovine rhodopsin template (1HZX) using the internet-based protein-modeling server, SWISS-MODEL (GlaxoSmithKline, Geneva, Switzerland), and energy minimized using the Gromos96 force field in DeepView. Extracellular and cytoplasmic loop regions were manually constructed, built according to JPred consensus, and energy-minimized using the NAMD molecular dynamics simulator.

### Biochemical and Pharmacological Analysis

Site-directed mutagenesis and transient transfections were performed on COS-1 cells as previously described [34]. Radioligand binding experiments (saturation and competition binding) [34] were then performed. IC_50_ values were converted to Ki using the Cheng-Prusoff equation, and values were expressed as a mean ± SE. For saturation binding experiments to determine Bmax and K_D_ concentration of [^3^H] SQ-29548 was varied from 1 to 100 nM. Nonspecific binding was determined by the addition of a 500-fold excess of non-radiolabeled SQ-29548. Exponential decay rate constants (k) and half-lives were derived through nonlinear regression using Prism. A 95% confidence interval was used for all curve-fitting procedures using Prism.

### [^35^S]GTPγS Thromboxane A_2_ Receptor Activation

Activity of hTP was measured by the accumulated incorporation of GTP analogue [^35^S]GTPγS (0.04 nM) into membrane preparations, following stimulation with 80 nM of synthetic TP agonist U-46619 (Sigma-Aldrich, St. Louis, MO). A concentration corresponding to the EC_50_ for aggregation of washed platelets [35]. Briefly, isolated membrane preparations were diluted in activity buffer (10 mM MgCl_2_ and 100 mM NaCl in 20 mM Hepes/Tris, pH 7.0) according to their BCA-measured protein concentration. An excess of guanosine diphosphate (GDP) was added (50 µM GDP) with or without addition of “cold” unlabeled GTPγs (10 µM), mixed with radiolabeled 5 nM [^35^S]GTPγs, and added to increasing concentrations of U-46619 in a 96 well plate. The plate was incubated for 2 hours at room temperature and quenched upon transfer to glass fiber filter papers with ice-cold 10 mM Tris-HCl. Filter-trapped radioactivity was quantified by liquid-scintillation counting.

### Patient Recruitment and gDNA Sequencing

Patients were recruited from Dartmouth and John’s Hopkins as previously described, under protocols approved by their respective IRB [36] [34]. Presented in Table S1 are the cardiovascular risk profiles for each cohort. Genomic DNA was extracted (EDTA-anticoagulated blood) using the Puregene® system (Gentra Systems, Inc.). Samples were Sanger-sequenced for isoform-independent Exon 2, and hTP-α and hTP-β Exon 3 (Genbank NM001060 and NM201636, respectively).

### Megakaryocyte Transfection and Flow Cytometry Measurement of Platelet-like Particle Activation

Transient transfection of Meg-01 cells (ATCC, Manassas, VA) was performed using the Nucleofection Kit C (Lonza, Walkersville, MD). The percentage of activated platelet-like particles was determined using two-color flow cytometry. Non-adherent cells and platelet-like particles were collected from megakaryocyte media 24 hours post Nucleofection, centrifuged at 1500 rpm for 10 minutes, and resuspended in PBS. Samples were treated with either 100 nM U46619 or equal volume of vehicle. Pelleted samples were resuspended, blocked with 3% BSA in PBS, pelleted again, and resuspended with phycoerythrin-labeled (PE) anti-CD42 and fluorescein isothiocyanate-labeled (FITC) anti-62p (P-Selectin) (BioLegend, San Diego, CA). Flow cytometric analyses were performed using a Becton-Dickinson FACSCalibur (Franklin Lakes, NJ). Twenty thousand events were recorded for each sample. In contrast to the *[^35^S]GTPγS Thromboxane A_2_ Receptor Activation described above, we were additionally able to accurately measure basal activity, in part due in part to signaling amplification.*

### Statistical Analysis

Data was analyzed using GraphPad Prism^®^ software (GraphPad Software, Inc., San Diego, CA). All means and SEM were calculated from at least three distinct experiments using separate protein preparations. Where applicable, statistical significance of the data was evaluated using an ANOVA and/or unpaired *t* test (*, p<0.05; **, p<0.01; ***, p<0.001). Analysis of variance (ANOVA) and student’s *t* tests were used to determine significant differences (p<0.05).

## Results

We hypothesized that hTP genetic variants may have a substantial modulatory effect on platelet activation.

### In Silico Analysis: Discovery, Modeling, and Prediction

The *in silico* analysis consisted of three major components. There was an initial search (NCBI dbSNP) for hTP genetic variant. A homology model of the hTP was then developed to assess variants to pursue. Predictive algorithms were then used to calculate whether the variants would be functional.

**Figure 1:**
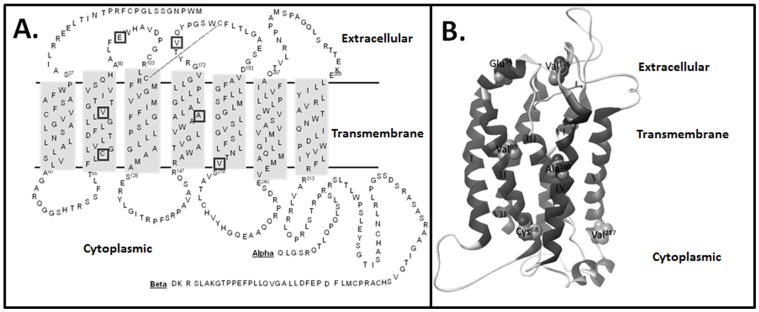
Structural Location of hTP Variants. **A.** A two-dimensional secondary structure representation of the hTP indicating the position of the six non-synonymous (squares) genetic variants studied; in the extracellular region (E^94^V and V^176^E), in the upper transmembrane region (V^80^E and A^160^T), and at the cytoplasmic interface of the lower transmembrane region (C^68^S and V^217^I). **B.** Three dimensional homology modeling of the hTP receptor demonstrates the relative positions of these intriguing variants to occur in the ligand-recognition loop (E^94^V), in proximity to the structurally critical disulfide bond (V^176^E), lining the putative ligand binding pocket (V^80^E and A^160^T), and in proximity to the G-protein coupling site (C^68^S and V^217^I). These variants were evaluated in detail by molecular biological, biochemical, and pharmacological techniques.

At the beginning of our study, we identified 17 nonsynonomous genetic variants within the coding region (exons two and three) of the human thromboxane receptor from the dbSNP (**Table 1**), only 9 occured within the isoform-shared exon 2. Surprisingly, alignment analysis (PAM250 similarity matrix: 30.16 versus 33.48, respectively) determined hTP to be more similar in sequence and structure to bovine rhodopsin (UniProt P02699) than the human beta2-adrenergic receptor (UniProt P07550). The seven transmembrane (TM) bundle generated by Swiss-Model had minimal structural deviation from the reference model (2.8Å crystal structure of rhodopsin (1HZX) after minimization. Based upon this model we selected 6 novel mutations (**Figure 1A**) putatively involved in ligand selectivity (E^94^V, V^176^E in the extracellular - EC domain), ligand binding (V^80^G, A^160^T in the transmembrane-TM domain), receptor trafficking, and G-protein coupling (C^68^S, V^217^I towards the cytoplasmic-CYTO domain) (**Figure 1A and 1B**) for further analysis.

**Table 1:** Non-synonomous Genetic Variants of the hTP Receptor from dbSNP.

mRNA position	Reference Nucleotide	Observed Nucleotide		RS#	Ref. AA	AA position	Variant AA	Isoform
202	T	A	5743		**C**	**68**	**S**	α/β
239	T	A	5744		**V**	**80**	**E**	α/β
668	A	T	5746		**E**	**94**	**V**	α/β
865	C	A	5749		**A**	**160**	**T**	α/β
914	T	A	5750		**V**	**176**	**E**	α/β
1036	G	A	5751		**V**	**217**	**I**	α/β
1235	C	A	201364793		**S**	**283**	**Y**	α/β
1311	T	C	4523		**Y**	**308**	**Y**	α/β
1338	G	A	201199706		**R**	**317**	**Q**	α/β
1373	C	T	201679561		**S**	**329**	**L**	α
1412	C	T	8113293		**T**	**342**	**M**	β
1439	T	C	34486470		**L**	**351**	**P**	β
1442	C	T	13306048		**P**	**352**	**L**	β
1460	G	C	5759		**R**	**358**	**P**	β
1495	G	A	191226440		**V**	**370**	**I**	β
1582	A	G	200445019		**T**	**399**	**A**	β
1601	G	A	10425128		**R**	**405**	**K**	β

Six commonly used predictive approaches were used to evaluate each of the six structurally diverse variants (**Table 2**). Interestingly, there were distinct discrepancies between some predictions (general disagreement between ProPhylER and PolyPhen2, as well as between SNPs3D and SIFT), but some were in agreement (Grantham and SIFT always agreed with each other, as did PolyPhen2 and MutationTaster) (Table 2). There was unanimous agreement amongst algorithms for only the V^80^E and V^217^I variants (Table 2).

**Table 2:** Predicted Effects of hTP Variants.

hTP-α Variant	Polyphen		Mutation Taster		SIFT‡	SNAP	ProPhylER§	SNPs3D	
	PPH	PPH2*	MT	Grantham†				SVM║	PSSM#
C^68^S	Benign	0.748	Disease	3.05	0.42	Neutral	0.9999	−0.05	−1
V^80^E	Disease	0.980	Disease	3.30	0.13	Non-neutral	1.29e-06	−1.42	−5
E^94^V	Disease	0.067	OK	3.30	0.32	Non-neutral	0.1624	1.43	0
A^160^T	Benign	0.660	Disease	2.92	0.19	Non-neutral	0.4032	1.01	0
V^176^E	Disease	0.721	Disease	3.30	0.27	Non-neutral	0.06689	0.08	0
V^217^I	Benign	0.000	OK	0.97	1.00	Neutral	0.9965	1.76	2

* PolyPhen-2 provides two results based on HumVar (13,032 human disease causing mutations from UniProt and 8,946 human nonsynonymous single-nucleotide polymorphisms (nsSNPs) and HumDiv [29]. These prediction algorithms return a scaled probability from 0 (neutral) to >1 (damaging).

† The Grantham score [32] represents the original amino acid substitution scoring method, presenting a scale increasing from zero to indicate the degree of side-chain physicochemical difference between the original and mutant amino acid. Single digit scores are considered conservative, with severe mutations ranging 60 or higher.

‡ The SIFT (Sorting Intolerant From Tolerant) algorithm (http://blocks.fhcrc.org/sift/SIFT.html) [26] infers functional importance from sequence homology. Based on a PSI-BLAST search alignment, SIFT returns a scaled probability matrix for the likelihood of a protein to tolerate each of the twenty amino acids at each position in the protein. Output values for each amino acid change tolerance from SIFT ranges from 0 (damaging) to 1 (neutral).

§ ProPhylER (http://www.prophyler.org) [31] combines the physicochemical properties of amino acid side chains and the observed evolutionary variation of those properties to infer deleterious substitutions.

║ The module of the SNPs3D resource (http://www.SNPs3D.org) [30] which focuses on predicting SNP influence on protein function, separately evaluates protein structural stability analysis and sequence conservation using a support vector machine approach trained on monogenic disease. Negative values are considered deleterious, positive values considered neutral, and values beyond ±0.5 indicate the degree of confidence.

# Position specific scoring matrix returns sequence alignment-based probability, with lower scores for damaging variants.

### In vitro Biochemical Analysis; Expression, Binding and Activation

**Figure 2:**
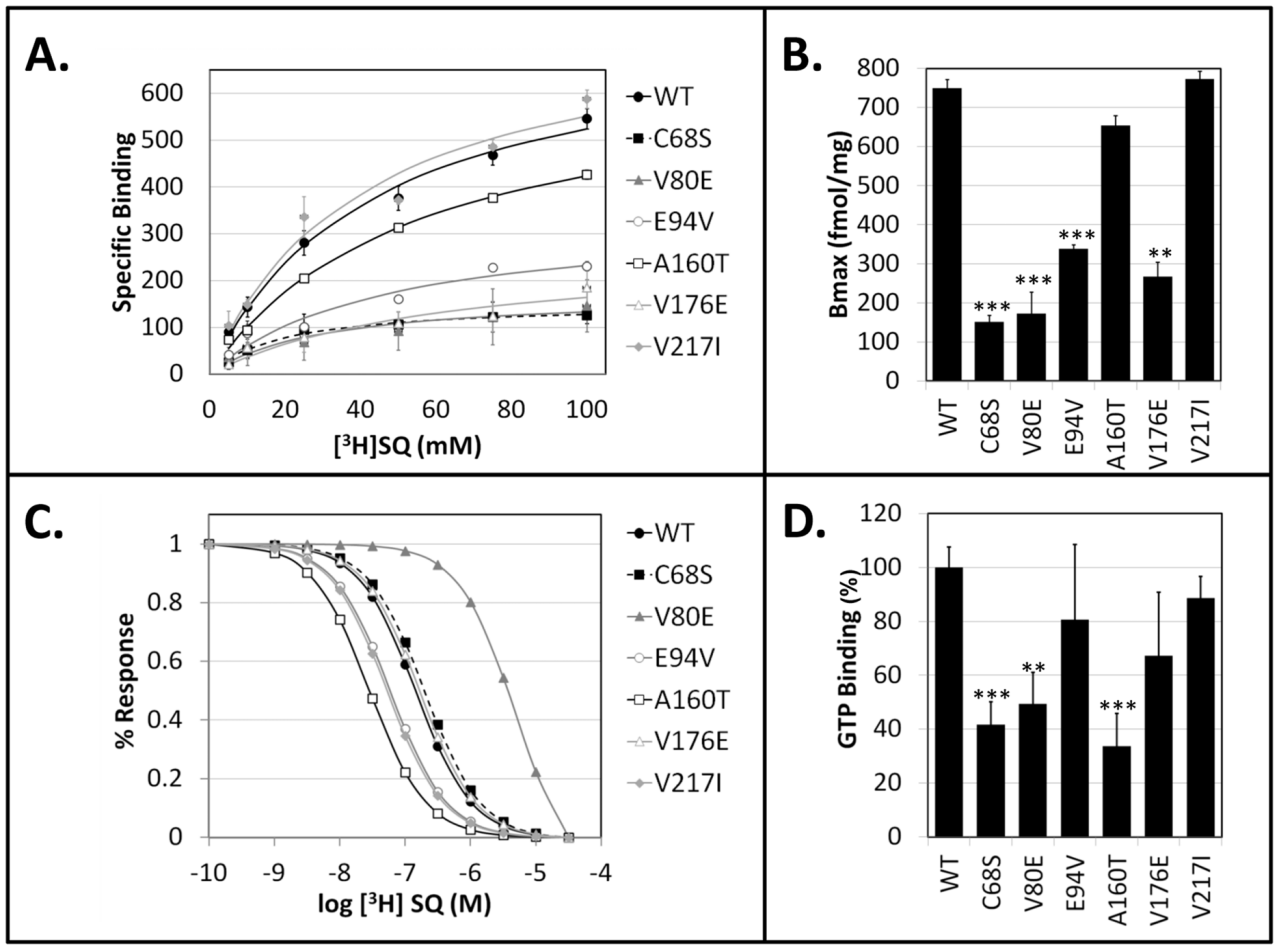
Competition and Saturation Binding Analysis. **A.** Saturation binding curves of hTP variants demonstrates a spectrum of binding deficits ranging from completely wild type (V^217^I) to severely dysfunctional (C^68^S). **B**. Histogram of the cell surface expression (Bmax) for each of the genetic variants analyzed. Significant differences from WT are highlighted (*p<0.05, **p<0.01, ***p<0.001). **C.** Competition binding of hTP variants demonstrates the relative affinity of antagonist for binding-competent receptors remains mostly normal. Statistically significant differences was observed only for A^160^T (increased affinity) and V^80^E (decreased affinity). **D**. GTPγS activation by hTP Variants upon treatment with TP agonist U-46619. Non-synonymous variants of hTP demonstrate different capacity to maximally stimulate effector signaling through GTPγs. The V217I, V176E, and E94V were indistinguishable from wild-type hTP receptor. Each of the other hTP genetic variants, C68S, V80E, and A160T, each demonstrate deficient effector activation with 80 nM U-46619 agonist stimulation. (*p<0.05, **p<0.01, ***p<0.001) Further details are provided in Table 3.

**Figure 3:**
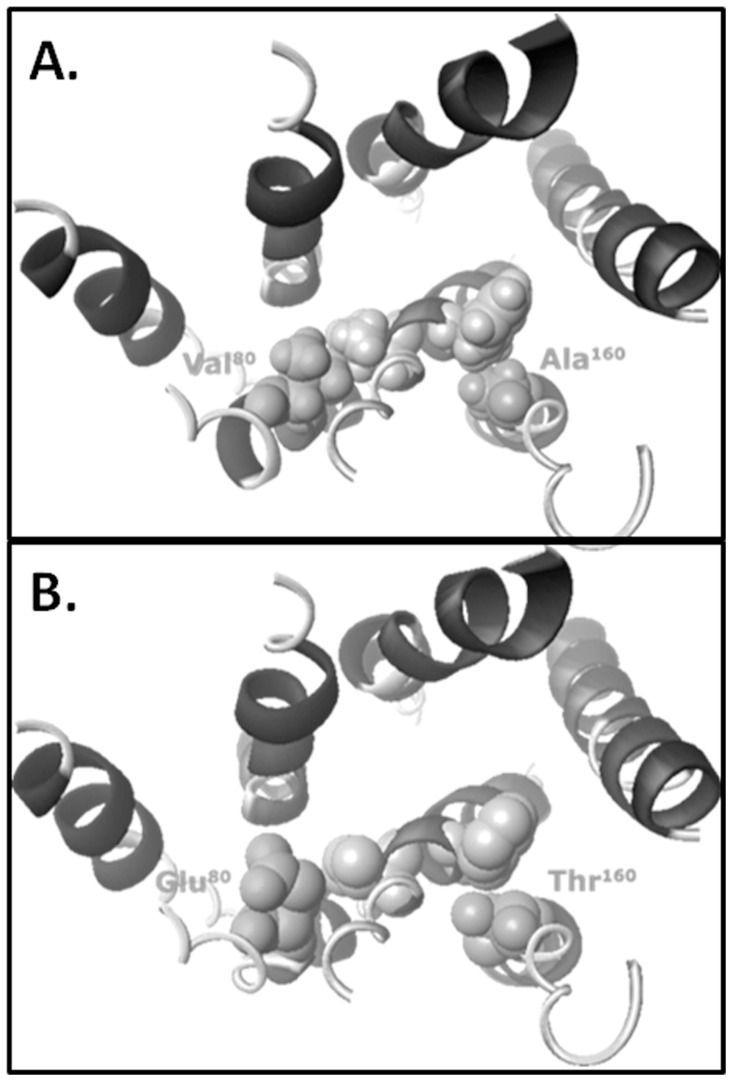
Binding Pocket Model of Disruption with Two hTP Variants. **A.** A model of the wild-type binding pocket. The hTP receptor structure from the extracellular surface illustrates the putative binding pocket formed between the alpha helical bundles in the wild-type receptor. **B.** Binding pocket changes observed with the variants V80E, and A160T. Alteration of residue 80 to a glutamate shows an obstruction of the binding pocket by the protrusion of a charged side chain into the channel. Changing residue 160 to a threonine, on the other hand, results in a loss of interaction in the hydrophobic cluster.

Saturation binding, with antagonist SQ-29548, provides a quantative profile of receptor surface densities (Bmax, fmol/mg). C^68^S, V^80^E, E^94^V and V^176^E were significantly lower than wild-type, whereas A^160^T and V^217^I were not significantly different from wild type receptor (**Figure 2A and 2B** **and** **Table 3**). Competition binding provides high resolution binding affinities. While most of the variants display binding parameters similar to wild-type, the variants localized to the putative ligand binding region of the receptor, A^160^T and V^80^E, displayed increased or significantly decreased affinity, respectively (**Figure 2C** **and** **Table 3**). Molecular modeling of these site modifications suggested that the change of residue 160 from alanine to a threonine, disrupted the helical packing with transmembrane domain three (**Figure 3**). The larger charged side-chain of Glu^80^ likely protrudes into the putative ligand binding pocket, providing an explanation for the observed reduction in binding affinity.

**Table 3:** Variant hTP Receptor Properties.

hTP-α Variant	Bmax (fmol/mg)	n	Log IC_50_ (nM)	Ki(nM)	n	GTPγS Activity (%)	n	Summary
WT	750.1±61.9	12	−6.71±0.19	5.87	11	100.0±7.6	7	Normal
C^68^S	151.4±27.2***	3	−6.74±0.27	2.90	6	41.7±8.4***	3	Unstable^#^
V^80^E	172.0±77.7***	3	−5.17±0.42**	154.49**	6	49.3±11.8**	3	↓ Affinity, ↓ Activity
E^94^V	338.8±38.7***	4	−7.24±0.08	1.81	5	80.7±27.8	3	↓ Accessibility║, ↓ Activity
A^160^T	654.2±63.3	3	−7.59±0.10*	0.91*	3	33.6±12.2***	3	↑ Affinity, ↓ Activity
V^176^E	267.2±110.9**	3	−7.09±0.33	3.14	3	67.2±23.6	3	↓ Accessibility║
V^217^I	773.1±78.5	3	−7.27±0.02	1.54	4	88.6±8.0	3	Normal

# Increased degradation as observed from Figure 4 however this does not exclude additional problems with transcription, RNA stability, or translation.

**Figure 4:**
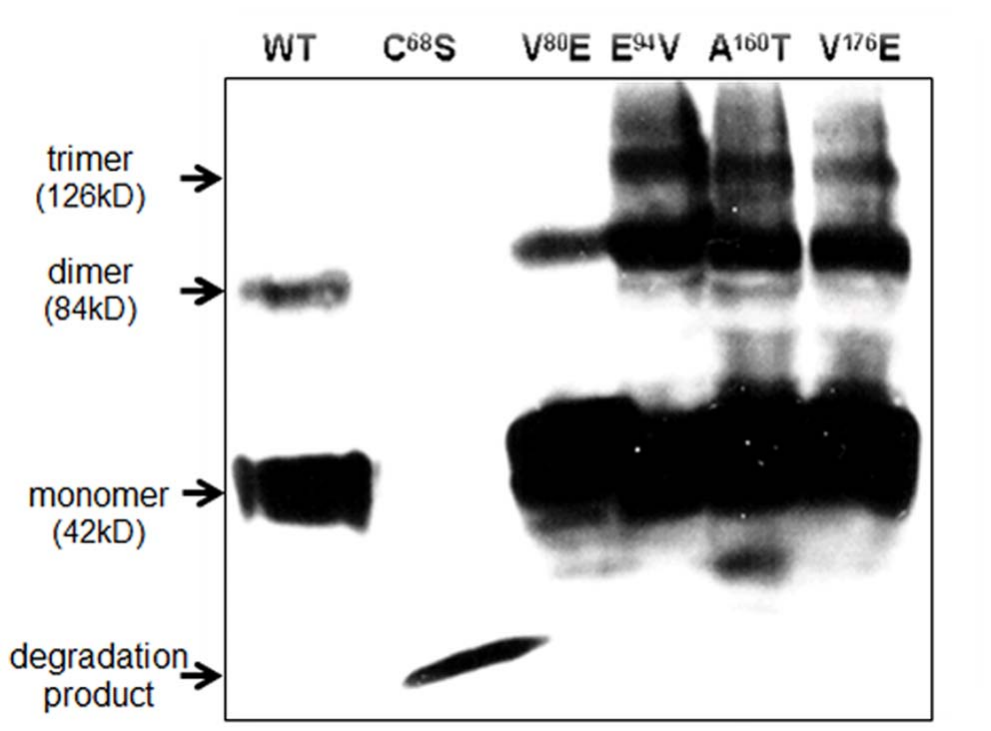
Western Analysis of Dysfunctional Variants. Western analysis for wild type hTP and the 5 dysfunctional mutations expressed in COS-1 cell membrane preparations. This was used to complement the saturation binding (folded protein) to assess for total cell surface expression (folded and misfolded). Monomers and oligomer formation is typical for GPCRs especially the misfolded variants. Shown are the approximate molecular weights in kD. The C^68^S demonstrated rapid degradation despite cocktails of protease inhibitors. This western analysis is representative of 4 performed.

║ Reduced accessibility refers to normal expression of cell surface protein (Figure 4) but a reduced ability to bind ligand.

* p<0.05, **p<0.01, ***p<0.001.

Although the various naturally occurring genetic variants of the hTP receptor demonstrated a spectrum of dysfunctional binding and expression, the crucial measure of function is the ability of the formed receptor to couple to its cognate G-protein. Notable significant reductions in activation were found between the wild-type hTP receptor and the C^68^S, V^80^E, and A^160^T variants (**Figure 2D** **and** **Table 3**). A subsequent western analysis of the 5 dysfunctional mutations demonstrated robust expression of all the variants except for C^68^S, which we believe is unstable and rapidly degraded (**Figure 4**). This does not exclude other mechanisms such as improper processing of the transcript or inhibition of other events in biogenesis (e.g. translation). From these results, each variant clearly had distinct and divergent patterns of biochemical and pharmacological characteristics as summarized in **Table 3**.

### Search for Dysfunctional Variants in Two High Thrombotic Risk Cohorts

Most dysfunctional variants are very rare, however we attempted to search for them in a high cardiovascular risk population. A high incidence of platelet rich arterial thrombotic events (strokes and heart attacks), can be found in such high cardiovascular risk populations with recognized atherosclerosis. We rationalized that we would find hTP variants in such high cardiovascular risk cohorts (hyperactivity variants having more disease and hypoactivity variants having less disease) and would be able to directly study receptor function in native platelets. We found three V^217^I (normal function) variants, and a number of synonomous variants (nucleotide change but no change in amino acid – **Table 4**) we did not find any of our dysfunctional variants in sequencing 897 high cardiovascular risk patients from two separate cohorts (Dartmouth and John’s Hopkin’s). These dysfunctional mutations may be protective against cardiovascular disease in a manner analogous to low dose aspirin (325 mg or less), which reduces thromboxane production and is cardioprotective. We excluded using a mouse model system as we and others have demonstrated that the thromboxane system in mice differs significantly from humans [36], [37], [38]. In the absence of direct patient samples we needed to develop a system to measure platelet activity in the presence of the genetic variants.

**Table 4:** Distribution of hTP Variants detected from sequencing two cohorts (n = 897).

hTP-α Variant	SNP ID	Codon	Position	Expected*	Observed
**T^81^T**	rs5745	ACC/ACT	c.243C>T	156	135
**S^145^S**	rs5748	TCG/TCA	c.435G>A	53	81
**T^186^T**	rs34881364	ACG/ACA	c.558G>A	19	5
**A^254^A**	rs5752	GCC/GCA	c.762C>A	27	2
**V^217^I**	rs5751	GTC/ATC	c.649G>A	41	3
				**296**	**226**

* Expected observation values were defined as the multiplication product of the general population frequencies reported in dbSNP and the number of samples measured in our patient population.

### “In Platelet” Functional Analysis

**Figure 5:**
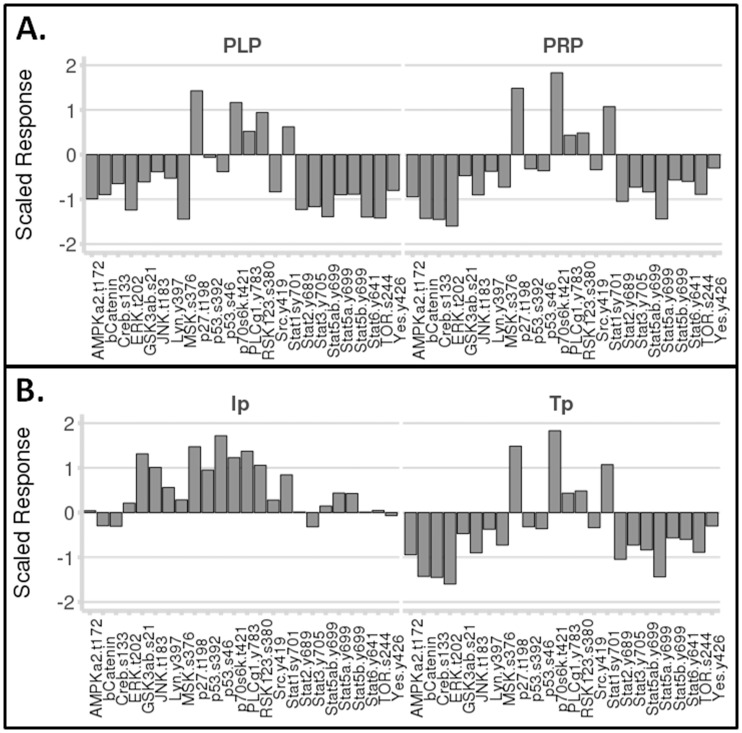
Development and Validation of Platelet Like Particle (PLP) System. **A.** Phospho-proteomic array demonstrating signaling of thromboxane response (U-46619 -100 nM) in PRP and PLP. Each of three arrays was performed in duplicate. **B.** Prostacyclin receptor activation with iloprost (100 nM) is compared to that of thromboxane (U-46619–100 nM) demonstrating distinct differences in signaling.

**Figure 6:**
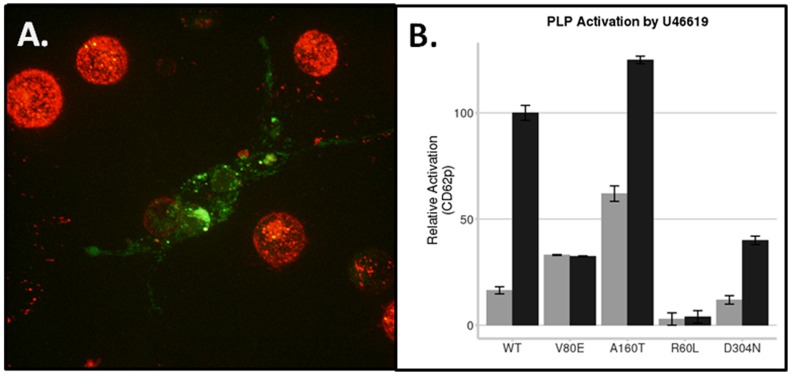
hTP Functional Analysis in PLP. **A.** Meg-01 Cells were nucleofected with hTP. Surface adherent megakaryocyte stained for p-Selectin (green) surrounded by produced platelet-like particles over-expressing nucleofected RFP-hTP-alpha. **B.** Stimulated display of CD62p (P-selectin) upon U-46619 treatment (100 nM) was reduced for the dysfunctional V^80^E variant, as well as completely unresponsive for the known bleeding variant R^60^L and D304N. Both baseline and stimulated levels of P-selectin were higher than wild type for the A^160^T variant, suggesting a considerable degree of constitutive activity. At least three independent repetitions were performed for each construct. Shown are mean ± SEM.

In the absence of patient samples in order to assess the downstream functional consequences, we needed a human platelet system that could be genetically modified. We discovered that a human megakaryocyte cell line (Meg-01) could be transfected and can produce platelet-like particles (PLP). We then verified whether thromboxane signaling in PLP reflected that of human platelets. We used a series of phosphoproteomic arrays to compare thromboxane signaling from PLP and human platelet rich plasma (PRP) (**Figure 5A**). There was remarkable similarity in the patterns of phosphorylation upon hTP stimulation (**Figure 5A**). As a control, prostacyclin signaling was clearly very different (**Figure 5B**). Our initial experiments with an RFP labeled hTP demonstrated abundant expression in the produced PLP (**Figure 6A**).

Individual variants that demonstrated signaling defects and were structurally stable (V^80^E and A^160^T) were then transfected into the Meg-01 cells and PLP collected for analysis. Wild type hTP transfection served as the negative control. As positive controls, we also reproduced two recently described hTP mutations that lead to a bleeding phenotype (R^60^L and D^304^N) [25], [39]. PLP were measured by flow cytometry for CD62p (P-selectin) exposure upon U46619 (thromboxane agonist) activation. Functional activation generally followed activity measured by effector coupling, with reduced activation by V^80^E variants (**Figure 6B**). The A^160^T variant, interestingly, showed higher baseline and stimulated activity than wild-type. This finding supports recent results suggesting a significant degree of constitutive activity for the A^160^T variant [40]. The V80E variant demonstrated dramatically reduced agonist response. Comparison of the V^80^E variant with known hTP bleeding variants (R^60^L and D^304^N) indicates that V^80^E retains some residual activity, and has a higher inherent baseline signal.

**Figure 7:**
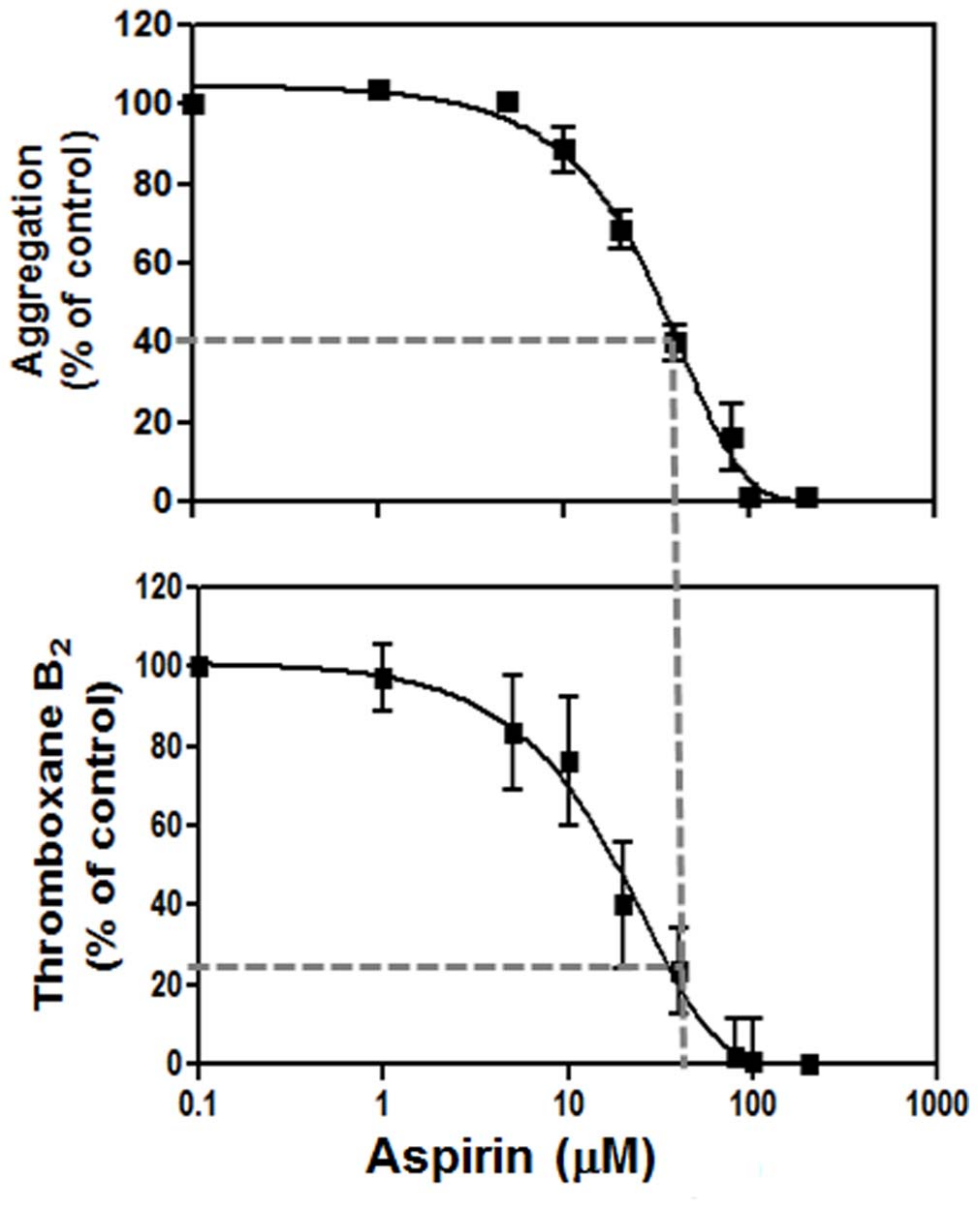
Analysis of Low Dose (325 mg) Aspirin Effect. *In vitro* aspirin dose response was performed for both aggregation and thromboxane production in human platelets (n = 4). The concentration equivalent to *in vivo* 325 mg aspirin (oral low dose aspirin) is indicated and the subsequent levels of thromboxane production and aggregation was calculated. This information was then used to determine which of the hTP mutation profiles best resembles that of “endogenous” low dose aspirin. This provides clinical and biochemical relevance to PLP results in the absence of patient samples.

In order to assess clinical relevance, we performed an *in vitro* aspirin dose response on thromboxane production and aggregation, using *in vitro* doses determined to be equivalent to *in vivo* daily low dose aspirin (325 mg) as determined by Pedersen et. al. [41] We concluded that 325 mg of aspirin inhibited thromboxane-induced platelet activation to a similar extent as the D^304^N mutation (**Figure 7**). This finding is clinically important as it demonstrates that a dysfunctional hTP variant can confer a degree protection from platelet activation similar to the effect of low dose aspirin therapy in our PLP model, suggesting there may be a similar protective effect against cardiovascular disease *in vivo*. This will assist in identifying protective genetic variants that serve as protective “endogenous aspirin”. The V^80^E is currently the closest to D^304^N. This may explain in part the paucity of dysfunctional hTP genetic variants in the cardiovascular population as such mutations are protective against disease. However, the A^160^T, with its increased activity, should be enriched in such a cardiovascular population. The likely truth however are that these are very rare variants, found only in specific populations. Further independent large cardiovascular clinical cohort studies are required to determine whether this is the case.

## Discussion

Surprisingly little is known about the influence of hTP genetic variants on receptor structure/function and atherothrombotic disease. With TxA_2_ being a powerful airway constrictor, major pharmacogenetic research has focused on the association of variants with severity and susceptibility to asthma [22] [23] and atopic dermatitis [24]. However two recently discovered mutations have been reported to be associated with bleeding diathesis; an R^60^L in the first cytoplasmic loop [25] and D^304^N, in the GPCR-conserved NPXXY motif [42]. More recently, the structural basis for dysfunction was described for A^160^T [40]. There have additionally been a number of manuscripts describing variants of thromboxane synthase [43], [44], [45], but no data for cardiovascular disease association. We set out to assess hTP genetic variants using a combination of *in silico*, *in vitro* and “in platelet” approaches. We encountered many issues that are impeding the clinical progress of pharmacogenetics, including 1) poor predictive values from commonly used predictive algorithms, 2) heterogeneity of biochemical phenotypes and 3) studying extremely rare mutations. However, our studies have provided new insights into the hTP structure function relationship, and have led to a new genetic approach to study “in platelet” function.

### Predictive Algorithms and Biochemical Phenotypes

With the enormous number of genetic variants that have been discovered, such algorithms, despite their limitations, can serve as a useful first pass analysis for detected variants; however, experimental receptor studies are mandatory. Variations in expression, binding, or activation require extensive in vitro testing. The functional defect can then be correlated to structural information. The development of such algorithms clearly needs to be informed by a team of structure/function experts in that particular class of proteins (e.g. GPCRs) in order to enhance accuracy.

Interestingly, each individual variant provided a unique signature of defects based in part on its localization within the protein structure in addition to the nature of the amino acid change. Such an understanding of the structural perturbation would be critically important for structurally based therapy. The A160T variant will likely require a negative agonist to dampen the enhanced basal activity. Furthermore, the affinity of the negative agonist would be required to be above that of thromboxane in order to effectively compete and prevent the functional hyperactivation. For V80E where binding affinity for thromboxane is severely impaired, possibly leading to bleeding, therapy may be needed to enhance thrombosis by targeting other receptors e.g. antagonizing the prostacyclin receptor. The C68S mutation may require ligands that help stabilize the receptor, preventing its rapid degradation. Thus, each mutation will require a distinctly different therapeutic approach.

### Studying Patients with Rare Mutations

All the nonsynonomous mutations studied are rare mutations found in only select populations, thus making it difficult to directly assess their functional defects conferred on native platelets. Our Meg-01 cell system can effectively characterize their function. This was particularly important for A^160^T which exhibited reduced GTPγS activation in comparison to WT; however, on a background of high basal activity in the Meg-01 system there was clear overall hyperactivity. The use of known patient mutations with bleeding disorders helped in validating our assay and the calculation of the effects of low dose aspirin also improved the clinical utility of such an assay.

There are many significant limitations with the current study, the foremost of which is the lack of patient platelets to study. We have tried to overcome this with analysis in a platelet like particle system. A further limitation is the modeling of our studies on the crystal structure of rhodopsin, another GPCR. Ultimately, the ideal modeling would be based upon a crystal structure of the thromboxane receptor. Finally, the resources to study the thromboxane receptor pharmacologically (as we have performed here) are now extremely limited as the ^3^[H]-thromboxane ligands are no longer commercially available.

Our study comprises the most comprehensive *in silico*, biochemical and functional evaluation of hTP genetic variants to date, and demonstrates a range of biochemical and pharmacological receptor dysfunction imparted by hTP receptor variants. Such cross-disciplinary studies are needed if we are to incorporate genetic variants into clinical patient management.

## Supporting Information

Table S1 Populations of High-risk CVD Patients sequenced.
(DOCX)
