# Correction: Human Thromboxane A_2_ Receptor Genetic Variants: *In Silico*, *In Vitro* and “In Platelet” Analysis

**DOI:** 10.1371/annotation/83ddfba7-3c48-4c96-8cd1-0f0b5f69a5d1

**Published:** 2013-07-30

**Authors:** Scott Gleim, Jeremiah Stitham, Wai Ho Tang, Hong Li, Karen Douville, Prashen Chelikani, Jeffrey J. Rade, Kathleen A. Martin, John Hwa

The name of the seventh author was given incorrectly. The correct name is: Jeffrey J. Rade. 

