# Supplementary material for: Human Thromboxane A2 Receptor Genetic Variants: In Silico, In Vitro and “In Platelet” Analysis
Source: PLoS One. 2013 Jun 28;8(6):e67314. doi: 10.1371/journal.pone.0067314 (PMC3696120; doi:10.1371/journal.pone.0067314)
Supplement: Table S1 — Populations of High-risk CVD Patients sequenced. (DOCX) [file pone.0067314.s001.docx]

## SUPPLEMENTARY Table:

**Table S1. Populations of High-risk CVD Patients sequenced**

|  | **Johns Hopkins Cohort** | **Dartmouth Cohort** | **Total** |
| --- | --- | --- | --- |
| **N** | 229 | 668 | **897** |
| **Male/Female** | 187/42 | 470/198 | **657/240** |
| **Median Age (Range)** | 63 (55-72) | 64 (31-92) | **63.5** |
| **BMI** | 29.5 (± 6.13) | 29.1 (± 7.7) | **29.3** |
| **Dyslipidemia** | 191 (83.4%) | 451 (67.5%) | **642** |
| **Diabetes** | 84 (36.7%) | 183 (27.4%) | **267** |
| **Hypertension** | 187 (81.7%) | 427 (63.9%) | **614** |
| **Smokers** | 52 (22.7%) | 108 (16.2%) | **160** |
